# The Hospital. Nursing Section

**Published:** 1905-01-21

**Authors:** 


### The Hospital.

**Nursing Section.**

Contributions for this Section of "The Hospital" should be addressed to the Editor, "The Hospital," Nursing Section, 28 & 29 Southampton Street, Strand, London, W.C.

No. 956.—Vol. XXXVII. *SATURDAY, JANUARY* 21, 1905.

### Notes on News from the Nursing World.

### THE RESIGNATION OF MISS WEDGWOOD.

We regret to announce that, entirely in consequence of family reasons, Miss Henrietta Wedgwood has resigned the post of matron of the Royal Free Hospital. It is now more than 20 years since Miss Wedgwood entered the Middlesex Hospital as a probationer. Shortly after the completion of her training she became sister at King's College Hospital. In December 1891 she was elected matron of the Royal Free Hospital, and under her auspices great improvements have been made at this institution in Gray's Inn Road, both in the training of the nurses and also in the accommodation provided for them. In 1894 the regulations were altered at the instance of Miss Wedgwood, and the period of training was extended to four years. In 1901 new quarters for the nursing staff, which had long been desired by the matron, were opened, and she was able to carry into effect her desire to supersede the system of cubicles, which she had always deplored, by a separate bedroom for every nurse. To her thoughtfulness and kindness the nurses are indebted for the possession of their comfortable study, which she furnished at her own expense. Miss Wedgwood has always been an advocate of the highest possible standard of nursing. Asked once whether she thought that there was a tendency to make the commercial element too prominent in nursing, she replied in the affirmative, and added, "Nursing was never intended to be merely commercial, and I deprecate any tendency in that direction. The principle of 'so much work so much money' is very good up to a certain point, but the work is not such that it can be done for money only without lowering it a great deal. Indeed, I maintain that it is utterly ruined if it is looked at from a purely commercial standpoint." Everyone knows how generously Miss Wedgwood has practised what she preached; and it cannot be doubted that the admirable influence she has exercised, quite as much as the ability and the tact which she has manifested during her term of office has augmented the reputation of the Royal Free Hospital as one of the foremost training schools in which nurses are not made to order, but taught the nature of their vocation.

### THE VACANCY AT THE ROYAL FREE HOSPITAL.

Applications for the post of matron of the Royal Free Hospital, which is now vacant, are invited. The salary is £100 a year, with board and residence. Candidates must be single or widows without encumbrance. They must not be under 30, and not more than 45 years of age. It is, of course, indispensable that they should be of good education, have received a full hospital training, and be able to undertake the teaching and training of the large nursing staff. Personal canvassing of the members of the board is not permitted. Application forms, and copies of the rules for the matron, can be obtained from the secretary of the Royal Free Hospital, Gray's Inn Road, to whom candidates for the vacancy should send in their letters, accompanied by not more than three recent testimonials, on or before February 11th.

### THE NURSES' HOME AT GRIMSBY.

The Committee of Grimsby and District Hospital, we are glad to learn, have held a meeting at which they promptly decided to act upon the recommendation of the chairman, Mr. C. F. Carter, and to revise the plans for the Nurses' Home now in course of erection. As the result of the meeting, immediate instructions were given to the architect to alter the plans, and the chairman writes, "We shall now, acting upon your suggestion, have bedrooms for the nurses instead of cubicles." It is very satisfactory to us to know that the committee unanimously decided to follow the advice of the chairman, and we think that the readiness with which they recognised the force of the arguments against cubicles conclusively proves that they are extremely anxious to do their utmost to provide for the comfort of the nursing staff.

### WOMEN INSPECTORS OF MIDWIVES.

The Norfolk County Council have decided by a majority of 43 to 19 to appoint a female inspector of midwives at a salary of £100 a year and travelling expenses. We are glad that the objections to the reasonable recommendation of the Sanitary Committee were over-ruled. It will now be interesting to see whether the new appointment is conferred upon a medical woman, or a nurse with general training in addition to her midwifery. We publish in this connection a letter from a midwife who thinks that the post of inspector of midwives might more fitly be given to midwives in actual practice. Her view of the matter is natural enough, but our opinion is that there are many reasons, including the fact that a great number of midwives have not been trained as general nurses, why higher qualifications than those of an ordinary midwife should be considered desirable. We therefore hope that the local authorities will continue to choose for the responsible position of inspectors women whose training enables them not only to see that a midwife discharges her duties properly, but also to note the presence of any abnormal symptoms which it might be of vital importance to have treated at once.

### PLYMOUTH GUARDIANS AND THEIR SUPERINTENDENT NURSE.

IN reply to the appeal of the superintendent nurse of Plymouth Workhouse Hospital for the reasons of the request of the Guardians for her resignation, she has been informed that they ascribe some of the frequent changes since she entered upon her duties in January 1901 to her high-handed treatment of the staff. The terms of the reply were agreed upon after a meeting of the Guardians at which Mr. Hawke declared that "the efficient work carried out at the hospital during Miss Halliday's term of office was due to her many excellent qualities. At any rate, she had seen that the nurses performed their duties, and there was not a single instance in which the sick had suffered through her management." This declaration had the effect of modifying the communication which the hospital committee proposed to send to her, but as it stands the allegation is of far too general a character, and the superintendent nurse will be fully justified in pressing for specific particulars.

### OLD AGE PROVISION FOR NURSES.

THE *Graphic* mentions that there is a proposal to establish a "Home for Decayed Nurses," and suggests that "a generous medical journal should make the matter its own, and, by achieving success, honour Miss Florence Nightingale, and assist the unfortunate in the nursing profession." But the settlement opened under the auspices of the Royal British Nurses' Association a few weeks ago, at Clapton Square, Hackney, and available for its members who are past work, was founded for that purpose. There are also the Benevolent Fund in connection with the Royal National Pension Fund for Nurses, and the Trained Nurses' Annuity Fund, which aim at succouring those older nurses who have reached a green old age.

### COMPULSORY CRADLES.

AT an inquest on Friday last on the body of a baby suffocated whilst in bed with the mother, Dr. Waldo said that it was useless to advise the poor to provide cradles, and that the only way to save infantile life was for Parliament to compel parents by law to do so. But before an act making cradles compulsory—which it would be most difficult, if not impossible to administer—is tried, the services of health nurses should be turned to account. Much can be done by quiet persistent advice and remonstrance, and if she is met with the objection that a cradle is too expensive to think of, the nurse who shows one mother how to convert a low, roomy, wooden box into a comfortable resting-place for the baby will, in due time, have plenty of imitators.

### A NEW OPENING FOR ELDERLY NURSES.

IT seems not unlikely that the success of the Council of the Guild of the Brave Poor Things, under whose auspices a craft school for cripple girls is shortly to be opened, may lead to developments which will be the means of providing suitable employment for elderly nurses. The matron of the craft school for girls, which will be conducted on the same lines as that of the school already established for cripple boys of the guild at The Heritage, Chailey, Sussex, is to be a skilled hospital nurse. The wisdom of appointing a nurse being recognised, we see no reason why the care of the health of cripples in this, or any other similar institution, should not devolve upon one who, while possessing sufficient energy for the work, and adequate training, has enjoyed long experience, and has learnt the art of patience in many spheres. A qualified elderly nurse should, in fact, make an excellent matron.

### NORTHAMPTON NONCONFORMISTS AND DISTRICT NURSING.

AT the annual meeting of the Northampton Town and County Nursing Institution, the chairman commented upon the fact that no Nonconformist place of worship in Northampton contributed to the funds, though a good deal of support was given by the churches. He attributed this to the erroneous idea that the institution is a Church of England organisation. Of course, it is nothing of the kind, and all the sick poor who require nursing receive attention, whatever their religion. We hope, therefore, that during this year the Nonconformist ministers will do their best to make up the difference of £50 which has been absorbed of the balance in hand of the District Nursing Fund, because the expenditure for 1904 exceeded the receipts by that amount. As regards the development of the work, 147 more cases were attended and nearly 2,000 more visits were paid. Miss Lunn, the indefatigable superintendent—whose private nursing department has increased instead of diminished the balance in hand of the institution—needs all the material support that can be given to her to enable her to make adequate provision for the needs of the patients. We are glad to see that the Guardians have increased their grant to sixty guineas, in recognition of the value of the work among the destitute poor.

### AN INCIDENT IN A FEVER HOSPITAL.

AN interesting case, around which there is more than a touch of sadness, has recently come under our notice. A young woman acting as nurse in a fever hospital in the Channel Islands, whilst on night duty last month had been feeling unwell, and, taking her temperature for herself, found that it was above normal. She consulted the matron, who gave her a dose of quinine and ammonia and herself rubbed her neck, which was stiff, with liniment. She was also seen by the doctor, who said that she might remain on duty. At the end of ten days the temperature dropped, but subsequently, when the nurse was dressing a child of three before the fire, she was taken with a fit. The child escaped injury, but the nurse received a slight burn on her chin. Three days later the nurse was informed that, having had an epileptic fit, she could not be retained on the staff. The authorities justify their action on the ground that, according to the nurse's own statement, she had three or four slight seizures of some sort between the ages of 12 and 14, and that if the medical officer had known this fact he would not have engaged her. It is alleged that he did not examine the candidate medically when he engaged her. But even had he done so he would not have known about the slight seizures several years previously unless she had told him of them. The relatives of the nurse complain that she was permitted to be on duty when she was manifestly unwell, with a rise in temperature, and her family doctor expresses the opinion that if she had rested when unfit to work, she would have had no seizure, as he did not hold the same opinion concerning the nature of the fit. Whilst sympathising with the probationer and her friends, we do not see that she has any claim to redress, though, of course, a nurse with a rise in temperature ought not to be on duty in the wards.

### QUEEN ALEXANDRA'S MILITARY NURSING SERVICE.

We are informed that Miss E. M. Goard and Miss E. J. Minns have been appointed staff nurses in Queen Alexandra's Imperial Military Nursing Service; that Miss E. Cox, Miss E. M. Monck-Mason, and Miss D. F. Palmer have resigned their appointments as sisters; that Miss M. L. Harris, sister, has been transferred from Woolwich to Chatham, and Miss P. Steele, staff nurse, from Royal Military College, Sandhurst, to Chatham.

### AUSTRALIAN ARMY NURSING SERVICE.

The following nurses have been appointed sisters of the South Australian branch of the Australian Army Nursing Service:—Mabel Dow, Mabel Alleyne, Alice McGregor, Mary Lord, Minnie McKittrick, Annie Cunningham, Fannie Herring, Elizabeth Bird, Mary Kelly, Ethel Davidson, Ada Webb, Elsie Buckhurst, May Townsend, and Ethelda Wren. They are all members of the Royal British Nurses' Association.

### MORE QUEEN'S NURSES WANTED.

There are openings at the present moment for hospital-trained nurses who desire to undertake district nursing under the most encouraging auspices. Applications should be addressed to the General Superintendent of Queen Victoria's Jubilee Institute, 120 Victoria Street, S.W., from whom all details as to rules and payment of Queen's Nurses can be obtained.

### THE AMERICAN CIVIL SERVICE EXAMINATION.

An examination has just been held by the United States Civil Service Commission with the view of selecting trained nurses for service on the Isthmus of Panama. The conditions of admission to the Service are interesting. The age limit is 20 to 35; the salary $50 per month, with board and quarters. Graduates of schools having a two years' course are admitted to the examination, and applicants having hospital experience in connection with the treatment of tropical diseases are preferred for appointment. The subjects of the examination are anatomy and physiology, hygiene of the sick-room; general, surgical, and obstetrical nursing, and experience in nursing. Only citizens of the United States are allowed to compete.

### STATE REGISTRATION.

A meeting was held at the Havergal Hall, Bournemouth, on Thursday last week to discuss State Registration for nurses, several of the leading Bournemouth medical men and every matron and lady superintendent of the local hospitals and "homes" being present, together with a large number of nurses and members of the public. Dr. Greaves was in the chair. Among the speakers were Miss Amy Hughes who dwelt on the ignorance of the general public as to the meaning of the word "nurse," and who said that it was no remedy for the present evils to suggest the registration of nursing homes and institutions; Miss Mollett who said that voluntary registration of nurses had been tried by the Royal British Nurses' Association, and that it had utterly failed; and Miss Annie Hobbs who gave details of various untrained nurses taking positions of grave responsibility for which they were totally unqualified. The Mayor of Bournemouth having congratulated the speakers on the eloquent and lucid manner in which they had proved the case for registration, Miss Helen Todd moved: "That in the opinion of this meeting the registration of nurses by the State is desirable as a measure calculated to promote their efficiency and as a safeguard to the public." The resolution was seconded by Miss Scott, and after a short speech by the chairman was carried unanimously. The speakers and the various matrons and superintendents of institutions present at the meeting were afterwards entertained to tea by Miss Todd at the National Sanatorium.

### HOSPITAL AND HOME FOR INCURABLE CHILDREN.

The Christmas treat at the Northcourt Hospital and Home for Incurable Children, Hampstead, took place last Saturday afternoon, when a large number of visitors were present. The entertainment consisted of a tea, carols by the children, and a concert arranged by Miss Holbyn. Miss Swinburne had coached the children for their carols, and Miss Lowe, for whom three cheers were given at the close of the treat, gave a ward tea. The wards were all nicely decorated by the nurses and children, and everyone present seemed to enjoy the entertainment. This is the first Christmas spent in the present building, and the committee are to be congratulated in having purchased this beautiful house, which was formerly the residence of Mr. Palmer, of Reading. The matron, Miss Forster, who is assisted by two Sisters, two staff nurses, and two probationers, is delighted with the change from Maida Vale, where the original Home was situated, and the 29 little inmates seem to appreciate the comforts of Northcourt.

### ENTERTAINMENT TO SOUTHAMPTON NURSES.

The medical superintendent and matron of the Incorporation Infirmary, Shirley Warren, Southampton, gave a successful "At Home" to the nurses on Tuesday evening last week. The nurses had permission to invite their friends. Some visitors from the town assisted in making up an enjoyable programme, which consisted of a little operetta, entitled, "The Feast of Flora." The costumes, representing flowers, were cleverly carried out. Others contributed songs and recitations. Altogether the nurses thoroughly appreciated the proceedings.

### NURSES AS WAXWORK FIGURES.

Last week a very successful entertainment was given at Derbyshire Royal Infirmary, by the members of the nursing staff, to the patients, doctors, and friends. After the performance of a one-act farce, "The Mere Man," several excellent tableaux were given by Sisters Yenking, McLoy; Nurses Bennett, Briggs, Dight, Dillon, and Gabitass. These were followed by some amusing waxwork representations by Sisters Simon, Keene, Yenking, Bosher; Nurses Mallison, Heelis, and Dorothy Yenking.

### The Nursing Outlook.

"From magnanimity, all fear above;
From nobler recompense, above applause,
Which owes to man's short outlook all its charm."

### NURSES AND SICK-PAY.

From the first day the Royal National Pension Fund started to the present, we have always felt that probably the most important advantage which it offered to the working nurse was sick-pay during incapacity for work. There can be no doubt that in the earlier days, at any rate, of a nurse's life, especially where she has been free from most of the ailments of childhood and youth, her calling entails a definite risk, greater than that run by workers who follow other occupations. So much is this the case, indeed, that the council of the Pension Fund had considerable difficulty in establishing the sick-pay branch upon the permanent and satisfactory basis which it has now attained to. The experience of 16 years has enabled the Council to evolve a policy which fully meets the requirements of the nurses, whilst it protects the policyholders as a body. The procedure is that sick-pay is granted only to certificated trained nurses, or persons attached to recognised hospitals or nursing institutions, and a certificate of training must be produced. A medical examination and a report by the nurse's own doctor have to accompany each proposal, which form has to be taken to the medical referee of the Fund or to one of its honorary medical officers. It is a little remarkable, seeing how much nurses have to do with doctors and the medical examination of patients, that a large number of them seem to have the greatest objection to submit themselves to examination at the doctor's hands. This idiosyncrasy will, no doubt, disappear more and more as the woman worker becomes accustomed to the regular pursuit of a definite calling in life. On marriage, a nurse must return her sick-pay policy for cancellation, though she may, if she wish to do so, qualify for a pension. Sickness assurance premiums are necessarily not returnable, though any nurse may enter for a pension on the returnable scale. A nurse may take out a policy for sickness assurance in the proportions of 5s. of sick-pay per week for each £7 10s. of pension for which she is a contributor.

We are aware that many nurses consider that if they are working for an institution there is no necessity for them to enter for sick-pay, seeing that at the best conducted institutions they will be taken care of, usually until they recover. This is generally true, no doubt, but a nurse may contract an illness of a permanent character, leading to prolonged or permanent disablement; or on her recovery from an acute illness she may need change and care in the country or at the seaside. Then, too, most nurses work for institutions when they are young—that is, at the time when it is most advantageous to enter for sick-pay because of the relatively small rates payable when a nurse is 25 years of age as compared with 35 years of age. Thus a nurse aged 25 can secure 10s. a week sick-pay for a payment of 6s. a year less than if she were to take out the same policy when she is 35. In the same way her premium for a pension at 50 years of age of £15 a year, the qualifying sum, would cost her £6 17s. a year more when she is 35 than it would do if she were aged 25. For this and other reasons the best friends of nurses—and who is a better friend than a gentlewoman who occupies the post of matron at a great hospital?—would render a very real service to the whole of their nursing staff, if they would take the trouble to explain these facts to their probationers during the period of their training at the hospital. The recognition of this principle at Guy's Hospital, the genuine kindness displayed towards the nurses by the staff there, and the refusal to sweat them, has resulted (through the federation scheme) in already entitling one nurse on the staff of the Guy's Hospital Nursing Institution to a pension of at least £60 a year. We hope that all certificated nurses will spend a little time upon this question of a provision against the day of sickness, and that the younger nurses, especially, will not lightly put it aside until they have mastered the advantages which the Pension Fund offers them, and have realised that at 25 for a payment of 23s. a year they can secure 10s. a week sick-pay, and so on in proportion up to £1 a week.

The younger nurse has another great advantage in this matter over her elder sister, because, as a matter of experience, the plan most frequently pursued is to issue a policy for three, five, or seven years, and if the policyholder enjoys good health it is then made permanent, when she can make herself secure under her double policy for the rest of her life. There are obvious advantages to a young nurse in entering early under a limited policy with a view to securing a permanent allowance when she takes her courage in both hands and commences nursing on her own account, for instance. Every year the nurses are availing themselves in large numbers of the free medical services of the medical referee, Dr. Potter, to whom the whole nursing body owe a debt of gratitude, not only for his generosity but for his genuine kindness to them during so many years. Many nurses who have no settled home and but few friends are coming more and more to look upon the Pension Fund as a haven where they can find security under all circumstances. It is difficult, as Dr. Potter declares out of his large and exceptional experience, to imagine a more really useful institution than the Pension Fund. It is, we know, a source of satisfaction, too, to the managers to find that so intelligent and meritorious a class of women as trained nurses now give the Pension Fund their entire confidence and also their very deep gratitude.

### Lectures Upon the Nursing of Mental Diseases.

By ROBERT JONES, M.D.Lond., B.S., F.R.C.S.Eng., M.R.C.P.Lond., Resident Physician and Superintendent of the London County Asylum, Claybury.

LECTURE XXIII. (*continued from page* 207)

An emergency which may readily occur in a certain class of insane persons, and with fatal results unless promptly relieved, is asphyxia by choking. It is most frequently due to an obstruction of the windpipe caused by some foreign body, usually pulpy food voraciously eaten, or some such substance as gristle or hard meat, which becomes impacted in the larynx, trachea or the bifurcation of the bronchi. Choking is more likely to occur in cases of general paralysis, epilepsy, in feeble and demented senile cases, and in those whose insanity—organic dementia—is the consequence of paralysis due to hæmorrhage or a blocking up of an artery in the brain, a condition described as thrombosis or embolism, and in whom the paralysis is, as a rule, on one side of the body. The place where choking most frequently occurs after bolting food is in the pharynx, the opening at the back of the mouth, which communicates with or opens into the nose above, the windpipe (larynx), and the gullet (œsophagus) below. Any foreign body which blocks the entrance to, or in any part of the windpipe, will cause choking. In normal swallowing the epiglottis behind and below the tongue covers the entrance into the windpipe, but food may pass into the windpipe when the epiglottis does not close over this opening, as may happen from obstruction caused by a large lump or a collection of food. Choking may also happen when a person speaks excitedly during a meal, as in chatty children, or when a person coughs or sneezes, or is seized with a fit during the act of swallowing food. A fish bone or false teeth stuck in the gullet is not choking, which occurs only when the windpipe is obstructed. In the case of an obstruction in the gullet the effort at vomiting may remove the foreign substance, and relief can thus be obtained by an emetic, or even by drinking water and rapidly gulping it down. Such obstructions may either be pushed down or brought up by means of "probangs"—special instruments passed down the gullet towards the stomach. The best remedy in cases of choking at meals is to deal the person a violent blow on the back between the shoulder blades, making him lean forward at the same time. If the obstruction is not at once ejected the finger is put into the throat, and an attempt made to clear it by hooking it up. If insensibility occurs and continues after the obstruction is removed, then artificial respiration is to be tried. If the obstruction is in the bronchi, and relief is not at once obtained, the patient may be "inverted" or held up by the legs or feet, and in all probability the medical man (who should be sent for at once) will perform an operation to admit air into the windpipe (laryngotomy or tracheotomy)—*i.e.*, an artificial opening into the windpipe from the front of the neck, and pass a long forceps into the wound to take out the obstruction. Assistance in cases of choking must be immediate, or life is lost.

Artificial respiration is an imitation of normal ordinary breathing, and can be carried out in three ways:—(1) Sylvester's method, when the nurse kneels on the floor behind the patient's head, grasping both arms below the elbows, bringing them up together above the head, by which movement air enters the chest, and then moving the arms down alongside the chest, or crossing them over the lower part of the chest, which is pressed in, thus causing air to be expired. This up-and-down movement is alternated about 18 times a minute. (2) Marshall Hall's method, when two persons roll the patient backwards and forwards, first on the face, then on the side, at the same time pressing in the chest in imitation of normal breathing. (3) Another method (Howard's) is to sit astride the patient, grasp the sides of the chest with both hands and press the chest in with some weight, squeezing the air out, and then rising, so that the natural elasticity of the chest-wall causes it to rebound, and draw in the air. These methods are applied to restore the apparently drowned as well as those suffering from asphyxia due to choking or narcotic poisoning, and must be kept up energetically for persons taken out of the water unconscious, for at least an hour—at the same time using every effort to keep up the natural warmth of the body. It is most important in all these methods to see that the tongue is pulled well forward and the jaw is depressed.

Not infrequently attempts at suicide with glass or cutting instruments occur among the insane; in such cases bleeding may take place, and may be so serious as to demand immediate attention. The best treatment is to take a clean handkerchief or a piece of shirt, linen, cotton-wool, or anything handy, and make direct firm pressure on the wound until assistance is obtained. If bleeding still occurs, pressure must be made on the "heart side" of the main artery, and if the bleeding takes place from a limb, this is to be raised. Bleeding of a serious character may take place from a varicose vein; in such an event pressure at the bleeding spot, as well as above and below this point, and raising the limb up well above the body lying flat, are the correct methods of treatment. Digital pressure on the wound is generally safe in all cases of bleeding, and should always be practised. In the case of wounds, until medical assistance arrives, wash and clean them with warm water previously boiled, and apply a clean handkerchief over them, or any soft article of wear washed in hot water.

In cases of burns and scalds there are three maxims to be observed. (1) Remove the clothes with care; (2) exclude the air; and (3) prevent collapse. Great care must be exercised in removing any clothing attached to the skin; indeed, it is often best to cut the clothes off; apply oil—usually *Carron oil*—composed of equal parts of linseed-oil and lime-water, with strips of clean rag, which should be well saturated with the oil; over these apply a good quantity of cotton-wool, and then a flannel bandage. Care must be taken to prevent shock by wrapping the patient in a warm blanket, and cautiously administering stimulants, and, if the pain is acute, a soothing opiate draught will be ordered. If oil is not at hand then use flour, whiting, or chalk made into a paste, and apply over the scald. If the skin is only reddened, the dressings may be soaked in a weak solution of bicarbonate of soda which will afford much relief to the pain.

Foreign bodies, such as stones, pebbles, beads, etc., are apt to be mischievously inserted into the ear. The best treatment is to syringe the ear, directing the stream along the roof of the ear channel and to pour in olive oil until skilled assistance arrives. If inserted into the nostril then a good sneeze may cause its ejection and removal. If in the eye, then turning the upper lid over backwards, what is called "eversion," or "turning the lid inside out," and lightly wiping it over with a silk handkerchief may remove fine dust or minute foreign bodies; or a fine camel's-hair brush may be used, also alternately opening and closing the eye under water—not an easy method with the insane—and, failing this, a drop of castor-oil may be placed in the outer angle of the eye between the lids until medical aid is at hand. All "drops" for the eye are put in at the outer angle, and with the end of the glass dropper resting on the lower lid.

If lime gets into the eye, wash immediately with weak vinegar and water.

As a rule injuries to the insane are of a serious character, and they themselves are also more difficult to treat in their injuries than those in full possession of reason; but such mild accidents as those above stated do frequently occur, and it is well for the nurse to be familiar with plain and practical remedies which are efficient as well as safe.

The next and concluding lecture will deal with emergencies accompanied by unconsciousness, with fractures, the care of the lying-in, and appliances in general use for sick nursing.

### Training Educated Women to be Nurses in Paris.

BY A CORRESPONDENT.

BEHIND the Pantheon, in a picturesque corner of old Paris, the pioneer training school for hospital nurses in France is quietly, but steadily, making its way and opening out a new career for educated Frenchwomen. The founder, Madame Alphen Salvador, began this much-needed but difficult work six years ago with three pupils, whom she established in a small apartment in the Rue Garancière, and for whom she obtained permission to attend the cliniques of well-known professional men at two of the large neighbouring hospitals. In a very short time more room was wanted, and the school was removed to a large, old-fashioned house at 10 Rue Amyot. The house is built round a courtyard, and part of it has been turned into a hospital, where six women patients may be received. The expenses of the hospital are met entirely by Madame Alphen Salvador, whose interest in the work never flags. The Training School is supposed to pay its own way by means of the pupils' fees, which are £32 a year, and the profits derived from the private nursing staff, the members of which are drawn from pupils who have trained in the School.

### CONDITIONS OF TRAINING.

The actual training extends over two years, in terms of nine months, from October 15th to July 15th. At the end of this period the nurse is asked to join the school staff of private nurses at a salary of £4 a month. If she refuses she must forfeit the sum of £40, and if at the end of the third year she decides to leave, £20, and at the end of the fourth £8. The training fees include lectures, board, and laundress, but not uniform, which is pink cotton for indoor wear, with the usual hospital apron, and blue serge, with cloak and bonnet, for outdoor. Each pupil has a room to herself, which she has to keep in perfect order, with the help of a servant once a week to polish the floor. Meals are served in a comfortable dining-room; breakfast at 7, lunch between 11 and 12, tea at 4 in the nurses' sitting-room, and dinner at 7. The matron and under-matron lunch and dine with the nurses, and Madame Alphen Salvador, when she is in town, generally joins them on Monday evenings. The off-duty times are not fixed, but are left to the discretion of the matron, who encourages each nurse to go out every day, and so arranges the work that it is possible for her to do so. No nurse is allowed out after 7, except on Sundays, when the time is extended to 9. The staff nurses may claim four clear days' rest in a month, as well as the regular yearly vacation.

### THE RULES FOR THE PUPILS.

Miss Scherrer, the matron, is the daughter of the late Monsieur Albert Scherrer, a Protestant pastor of some note, and a man of literary and political distinction. She has had some experience of nursing in London. The rules she has drawn up for the pupils are as simple and few as possible, but must be rigorously carried out. The first three months of the training are spent in the little hospital attached to the school, where, under the direction of Mademoiselle Guyot, an old pupil who has been promoted to the post of "directrice" of the hospital, the probationer learns the same lessons in cooking, cleanliness, and elementary nursing as does a probationer in any London hospital during the same period. At the end of the three months she passes on to the big public hospitals, where, through the influence of the visiting physicians and surgeons, the Rue Amyot pupils are allowed students' privileges, and assist at operations, do dressings, and get practical experience in medical nursing. Added to this, lectures, which may be attended by outsiders on the payment of a small fee, are given at the school four times a week by some of the best known men in the French medical profession, and by some of the Sorbonne professors on subjects relating to nursing, such as sociology, etc. At the end of the first year the pupils must pass an examination, and at the end of the second, a final. In the case of a candidate failing, another opportunity is offered before a special jury. Certificated nurses are employed at a hospital in the Rue Oudinot in connection with the School, and is is here that they get their best surgical experience. The private staff is now considerable, and many of the greatest Parisian doctors show their appreciation of having educated women to nurse for them by calling on the Rue Amyot school for their nurses in preference to employing the untrained, unlettered women who have up to now worked for them.

### THE ARRANGEMENTS OF THE HOSPITAL.

One of the most admirable points in the School is the clever way in which every corner of both the home and the hospital has been turned to the best account. The arrangements of the latter particularly show a talent for economy and adaptability which is most praiseworthy, for the house is full of odd corners and winding stairs. Each patient has a separate bedroom, simply furnished and scrupulously clean. The kitchen and bath-room are on the ground floor, and the former has to serve as out-patient room. The entire work of the hospital is done by the pupils, even to the cleaning of the black and white tiled floor in the kitchen, and by the general order which reigns throughout it is easy to see that the discipline, if not severe or ceremonious, is, at least, efficient. One morning a week the women in the neighbourhood, which is a very poor one, bring their babies to be weighed, fed, and examined; so that in this way the nurses get to know many of the neediest families, and as they are allowed to do a certain amount of district nursing on their own account, some of them have started funds for the benefit of those who are in the worst straits, and they are thus able to relieve a good deal of local misery.

### THE NATIONALITIES REPRESENTED.

A kindly spirit of tolerance pervades this quiet corner of the great French capital, and makes it possible for women of all nationalities and religions to work together happily. At present there are French, German, Italian, and Dutch pupils in the School. The majority of the French girls are either Protestants or orphans, for in a country where no well brought up girl is allowed to go out unattended either by her maid or her mother, such a necessarily independent career as that of a nurse is not looked upon with favour by the heads of families, especially if they belong to the Roman Catholic faith which associates nursing only with the purely religious life and exacts that all worldly pursuits be given up. With the Protestants the religious question interferes in a less degree, but the social side of the matter is just as serious, and there are few French mothers who would not look with distinct disapprobation on the idea of their daughters becoming "infirmières." The name alone is enough to frighten them, connected as it is so far with the class of women who act as nurses in the public hospitals since the "Sisters" were removed. The only qualifications necessary to join the School are a certain fixed standard of education and satisfactory personal credentials. Whether a girl be the daughter of a long line of gentlemen, or the educated daughter of a concièrge, is of no account. She is taken on her own merits and not those of her family, and apparently the system answers very well. The gentleman's daughter learns to appreciate the capability of the porter's daughter, and the latter, in her turn, catches something of the meaning of "noblesse oblige" and acquires a little of the quiet tact and dignity which Monsieur Lepage, in his lecture to the pupils on the beginning of this year's term, so strongly recommended to their notice. It was pleasant to hear Dr. Lepage speak enthusiastically of the London hospitals and the English nursing system. The difficulties of these pioneers are many, but with all the proverbial pluck of their race they are making a fine fight for their cause, and in spite of limited means and social and religious prejudices, they are making obvious progress. Those who know the existing nursing system in the big public hospitals of Paris will appreciate the importance of the struggle and the efforts of those professional men and women who are doing everything in their power to further the work and make it known.

### Beyond the Seas: a Visit to the Somerset Hospital, Cape Town.

The Somerset Hospital stands in a quite ideal position, on high ground at the very edge of the coast, overlooking Table Bay, so that the patients lying out on the broad stoeps can watch the great liners sailing in and out of the docks and can observe the movements of all the smaller shipping craft. It is a very fine imposing-looking building, situated in the midst of spacious grounds, where there is ample room for expansion.

### The Training.

Miss Child, the matron, was away on the occasion of my visit, but Miss Paul, the assistant matron, gave me every possible information. The nursing staff consists of a matron, assistant matron, night-superintendent, 12 ward sisters and 40 nurses and probationers. Nurses take a three years' course of training. During the first year lectures upon practical nursing are delivered by the matron and on sick-room cookery by the assistant matron. These last are considered most essential in South Africa, where a nurse may often find herself in a house where the cooking is all in the hands of a more or less competent Kaffir. The second year lectures are given by one of the resident medical staff and the third year's by the senior medical officer, Dr. Moffat. At the end of their three years' training, the nurses have to pass the Cape Government Medical Examination, of which two are held every year. It is hoped that the Medical Council will shortly pass a regulation requiring the three years' training to have been consecutive, at one hospital only, and not, as has hitherto been the case, a year at one and a year elsewhere and so on.

### Plenty of Applications.

No difficulty is experienced in obtaining nurses. The sisters have in nearly all cases come out from England, but the probationers are Colonial, some being Dutch, which, as a certain proportion of the patients speak only that language, is very convenient. Day nurses have breakfast at 7.30, and there is an interval at about 10 A.M. for lunch, dinner at 1, tea at 4, supper at 7. Each nurse has three hours off duty every day, and once a fortnight has what is known as "long pass," which means, from 2 P.M. for the rest of the day, up till 10 P.M. The night nurses go on duty at 9 P.M., and are off at 8 A.M. They generally do duty for three months, sometimes for less.

### No Home.

There is an urgent need for a Nurses' Home. A very brief survey sufficed to convince me that the present nurses' quarters are inadequate and unsuitable, especially in regard to the sleeping accommodation, which consists entirely of cubicles, several containing two beds. There is an excellent site in the grounds adjoining the hospital. Plans have been drawn and a certain amount of money raised, besides £3,000 which had been granted by Parliament. All that is required now is the consent of the Government to building operations being undertaken. The Somerset Hospital, although almost entirely supported by voluntary subscriptions, receives a yearly grant from the Government, and times have been so bad lately in Cape Town that the Government have hesitated to sanction any further outlay at present. However, the Public Works Department has submitted plans for proper nurses' quarters, and it is hoped that the building of the home will be begun shortly. But hope has told a flattering tale in this respect so often that it is not to be wondered that those most concerned should feel a little sceptical as to the fulfilment of these promises. About three years since the Princess of Wales laid the foundation-stone of this home, on the occasion of her visit to Cape Town, but the home itself is as yet no nearer realisation. The plans have been modified in order to lessen the initial outlay, and arrangements have been made that 15 of the nurses should still sleep in the hospital. The space now occupied by the nurses' quarters would be of enormous value to the hospital for an out-patients' department, which is badly required. At the present time the out-patients have to wait in the entrance hall. Another much-desired addition to the hospital is a laundry, as the washing at the present time has all to be put out.

### Existing Accommodation.

Having listened with much sympathy to an account of this unsatisfactory state of affairs, I was conducted over the hospital and was much interested to observe the points of difference between a South African and an English hospital. Whatever may be said of the sleeping apartments, the nurses' dining-room is certainly a very fine room. It was originally a children's ward, and with its many windows it looked extremely bright and airy. It contains a piano. The nurses have a nice cosy sitting-room, resplendent in fresh upholstery, but somewhat small for the purpose. After a peep at the sisters' pleasant-looking sitting-room, we passed on to visit the wards.

### The Wards.

The hospital contains somewhat under 200 beds, and has 12 wards in the hospital, and one male ward in the grounds for contagious diseases. All but one of these have a broad stoep along one side, upon which most of the patients lie or sit during the daytime, and some are there both day and night. The ward which has no stoep is to have this want supplied when the nurses' home is built, and all the attendant improvements take place. All the wards present an attractive appearance, with their wide-open windows, or rather doors, and bright fires, so cheerful to English eyes. A splendid show of flowers adorned the centre tables, all grown in the grounds I was told. There were arum lilies in profusion, bunches of pink-and-white heather, and great boughs of mimosa. The children's or "Lady Loch" ward, which is its right name, is very gay with hand-painted panels. Most of the small people were out on the stoep. In this ward the white and coloured children are together, and the black curly mops, bright eyes and brown skins of the native children form a good foil to the fair hair and pale cheeks of the European children. In one of the men's wards representatives of all nationalities were to be found; indeed, the sister in charge stated with some pride that she had both a Russian and a Jap under her care. Asked if she were not afraid of disagreement between the two rivals, she answered in the negative, because, she added, neither knew the other's language, and, moreover, the Russian was a Jew, and little likely to stand up for his persecutors. The residents of Cape Town are very kind in keeping the hospital well supplied with books, illustrated papers, etc., and on the last Saturday in every month a concert is given for the entertainment of those who are well enough to enjoy it.

### A Day in a Dispensary.

BY A LADY DISPENSER.

In a dingy road in a very unaristocratic part of London is a gloomy-looking old red brick house, surrounded by tall tenement buildings inhabited by many very poor families. This is a dispensary to which many come daily for advice and medicine, whose annual number of applicants runs into many thousands, bringing the daily average into three figures. If one pauses a moment how terrible it is to think of the suffering and trouble this means to many and many a family! In a well-ordered household in comfortable circumstances remember the anxiety and worry which a slight case of illness entails even to those who can afford the medicine and luxuries an invalid requires; but how far worse when illness strikes one down in a room inhabited perhaps by the whole family? The poor patient lies in a room with the odour of cooking, the wailing of a puny infant, and the hundred and one noises which come up from the street below, the windows generally tightly shut, or else broken and stuffed with rags or paper, thereby letting in draughts, all of these drawbacks combining to give the sufferer a greatly diminished chance of recovery. Small wonder then that the stricken ones struggle up from a sick-bed far sooner, under the circumstances, than they should.

### Patients for "Repeats."

The dispensary is opened at eleven o'clock, the doctors coming about twelve; but long before that time patients begin to arrive to get "repeats." Each patient is allowed to have the medicine repeated once without seeing the doctor. "Please can I have another bottle of corf medicine for the biby?" or "Muvver was took very bad in the night, please can you give 'er somefing?" "I've lost the bill (*i.e.* prescription), but can I have the same medicine as last time as it done me good?" are some of the requests of small people who imagine that the dispenser can remember what each patient has had each week, or can prescribe something for "Muvver who was took bad in the night;" quite regardless of the fact that the dispenser is not a doctor, and also that nothing whatever is known of "Muvver," who may be suffering from scarlet fever, or small-pox, or from paying too long a visit at the "Arms" close by overnight, or who may have simply a bad cold in her head.

### The Duties of the Dispenser.

Meanwhile, the room where the patients are waiting to see the doctors is getting gradually filled up, and the cries of wailing infants and impatient children fill the air. Each patient takes her seat in the order in which she comes in, so that the earliest comers see the doctor first; the men occupy a space reserved for them at the other end of the room from the women and children. The dispensers in the meantime have plenty to do besides attending to the wants of those who have come for repeats; cold infusions have to be made, the kettle put on to boil for hot ones, benzoated lard and white paraffin to be weighed out for the ointments which have to be made, bottles to be filled, shelves to be dusted, and every now and then orders to be written out for the wholesale druggist. Stock medicines and solutions have to be replenished. Towards twelve o'clock the doctors come, the resident medical officer generally getting in from his rounds before the other doctor arrives, the latter sometimes being very late. There is a stir among the patients, many of whom have come to see the resident medical officer, while others are waiting to see the visiting doctor. "Ah! he's a good 'un, he is," says a woman to her neighbour, nodding her head towards the doctor who is disappearing through his consulting-room door, "he saved my gal when I fair thought it was all up with her." "Did he, though?" answers her friend sympathetically. "Aye, he did that. Why, look at her now, she's a different creature to what she was a little while since." "Yes," says another woman, "and look at my boy; you know how he was, and he's that fat and well now, all owing to that doctor, bless 'im." "Now, then, which are you for?" breaks in the porter's voice, "for this room or that?" pointing to the consulting-room doors. "Oh! I'm for this one, o' course; d'ye think I'd go to any other doctor?" "Well, come along then. Make room there, please; move up, your doctor hasn't come yet," to another. In a minute the little bell sounds, and the patients, one by one, ushered by the porter, go into the consulting-room, the men first, the women and children after, coming out again after a minute or two armed with a prescription which they take to the dispenser, to whom they hand it through a little window into the dispensing-room, receiving it back soon after, together with a week's or half a week's medicine, as the case may be. Presently the other doctor comes, and then the dispensers find their work cut out for them, the patients coming up for their medicine quicker than they can be attended to; the men, besides seeing the doctors first, are allowed to receive their medicine before the women, so as to enable them to get back to work, it being the dinner-hour of most of those who are not of the "unemployed."

### Making Up Prescriptions.

All, however, are attended to as quickly as possible; some have longer prescriptions than others, although a great number have stock medicines, which are absolutely necessary with such a number of patients coming every day—some of the children want, or rather need to have, powders, which take some time to weigh out; occasionally, too, a delay is caused by an illegible prescription or word which has to be sent back to the writer with a request for clearer writing! If would-be doctors could be made to pass an examination in legible handwriting what a boon it would be to some long-suffering dispensers! After a time the men are finished with, and attention is turned to the women, who have been patiently (or impatiently) waiting their turn. "Here, young woman, can't you give this lady her physic," says one woman, good-naturedly shoving another one in front of her, she wants to get home, her baby's ill; I can wait." All the patients call each other "this lady" or "that gentleman," while the porter who attends to them and gives them their tickets is referred to as "the gentleman what sells the tickets." "No, it wasn't you as served me last week, it was the other young woman; can't you give me the same physic again?" says another who is not pleased at finding her medicine changed. Gradually the time passes, patient after patient handing in her bottle and prescription. Many and various are the bottles brought, each woman being obliged to provide her own; sometimes a child will bring a quart bottle for five or six ounces of medicine; others will expect a pint of medicine to be put into a six-ounce bottle.

### How Some Patients Take Their Medicine.

Often a patient, or a patient's friend, takes a drink out of the bottle as soon as it is handed to her, utterly regardless of the directions on the bottle as to how and when it is to be administered. One day a little boy, looking terribly pale and ill, came with his mother; she was told to take him home and put him to bed, and was given a week's medicine for him. The medicine is usually given to adults, but the child was told to take a teaspoonful three times a day instead of the usual larger dose. Next day the woman came again for more medicine. "What have you done with what you had yesterday?" she was asked. "Oh! I've given that to the boy," she replied. "Do you mean to say you have given him all that? A week's medicine in a day!" was the amazed question. "Yes, wasn't that right? I sat up all night to give it him. Well I never!" Whether the boy recovered or not remains unknown. After a time the last patient has been attended to, the doors are shut, and the dispensers are free to sit down and talk over the day's work while they have their lunch. As soon as they have had a short rest they again set to work, tidying up, washing infusion pots, cleaning ointment slabs, refilling the emptied bottles, and making up a few more "stocks," leaving the more unimportant ones until the following morning. Then the day's work is done, and the dispensers are ready to go home, after carefully locking up the cupboards and the poisons, so that no one can get in during their absence and help themselves.

### Everybody's Opinion.

### WOMEN ON HOSPITAL BOARDS.

Miss E. L. C. Eden, the Grange, Kingston, Taunton, writes:—In your comment on Lady Hermione Blackwood's letter you appeared to imply that all women were amateurs or devoid of special knowledge. In my letter the following week I ventured to challenge this opinion, but you let the glove lie. This week, in your note to Miss Jenner's letter, you again express the same views, as if no one could hold any others. Would you, perhaps, give your reasons for classifying the whole of a sex, which has produced not only Florence Nightingale, but Octavia Hill, Mrs. Fawcett, Agnes Jones, Mary Somerville, Mrs. Booth, Miss Weston, and many others under the one head of amiable butterflies?

[Our position is that all women who have not been trained to the work, and who have had no opportunity of acquiring special knowledge of hospitals and their management, are amateurs, and could not in fact prove useful members of a hospital committee. The business of a hospital is very important, and business men on hospital boards are responsible for this business. Technical matters are referred to the medical staff, or to the able ladies in charge of the departments where women are employed, who advise the board in all such matters, and in this way the highest efficiency can be maintained. What useful purpose could a woman, who has no knowledge of business or of technical matters applied to hospitals, fulfil, if she were to be elected a member of a hospital committee?

The names quoted by Miss Eden illustrate our point and enforce it. Miss Florence Nightingale and the late Agnes Jones devoted their lives to the study of hospital questions, especially in the departments where women with knowledge can usefully aid. They would no doubt have always been welcomed on a hospital committee, because they were technically qualified to serve. In the same way, Mrs. Fawcett would be welcomed as an expert in educational matters, and Miss Weston where the well-being of sailors is concerned. Again, Octavia Hill, for instance, would have been invaluable in matters relating to the housing and well-being of the poor, Mrs. Booth in all things affecting the social and spiritual well-being of the people, and Mary Somerville in certain branches of science, especially physics. The best hospitals are, in fact, managed, so far as the work which women can properly undertake is concerned, by ladies, the successors of Miss Florence Nightingale and Agnes Jones, who have acquired special knowledge for the discharge of their responsible duties by years of hard work. Does Miss Eden wish to maintain that any woman, because she desires to be upon a hospital committee, who has never had any special training, and who possesses no technical knowledge or experience of business would be able, if elected, to render any useful service at all? We are convinced she could not, but experience shows her ignorance, if accompanied by indiscretion and over-zeal, might work much mischief, which the hospitals at present are free from. That is why the more intimate the knowledge, the more certain is the conviction, to an independent mind, that no amateur ought to be elected to serve on the committee of a great hospital.—Ed. The Hospital.]

### WOMEN INSPECTORS OF MIDWIVES.

"W.," writing from Ireland, says: A recent number of The Hospital deplored the inadequate supply of midwives in England, and ascribed it to the very low salaries offered for midwifery. I have not the article by me to refer to, as I send my Hospitals away, so you will pardon me if I make a mistake. I should like to call your attention to another cause which will probably deter educated women from entering the ranks; that is, the want of any prospect of promotion. I daresay it is unavoidable, but in advertisements of any important midwifery post general nursing is always made an essential in addition to midwifery. I can see the advisability of this in hospital work, although I am sure you will agree with me that it is going a little too far when, a few years ago, a lying-in hospital advertised for a matron, saying, "midwifery not essential." Now, however, an opportunity has arisen for the promotion of educated midwives of good standing, in which general training is quite superfluous, but the opening which we have a right to expect is not given to us, and there is very little encouragement to good class women to enter a calling to the upper ranks of which we cannot expect to rise. I allude to the appointment by the County Councils of women inspectors of midwives. I have followed all the appointments with great interest, and have myself applied unsuccessfully for several. In every case the post has gone to a medical woman, or to a nurse with general training in addition to her midwifery. Considering what a very badly paid, and very trying life a midwife's is, would not it be only just that these good appointments should be reserved for midwives in actual practice, who surely might be expected to understand thoroughly the various weak points of their class, and what faults to look for, and who can give encouragement and instruction from their own actual experience? There are many posts for which a general nurse is suitable, and for which one with only midwifery training is not, but if her education and other qualifications are equal, one whose attention has been given exclusively to midwifery for, say, 10 or 12 years, should be better fitted as an inspector of midwives than a nurse who has divided the same length of time among several branches of nursing, or who has merely passed the L.O.S. examination, and has had no further practical experience. Of the action of a County Council in advertising for midwives with "practical training," and then giving the post to a lady doctor, I will only say that if medical women are considered eligible for the appointment the advertisers should say so, and spare busy midwives the trouble and suspense of competing with members of a superior profession. I have been midwife of a large district for six years and have attended successfully during labour and convalescence nearly 600 confinements, and a large number of other gynæcological and obstetric cases. I have never lost a patient nor had a case of puerperal sepsis in my practice, and I hold the very highest testimonials as to efficiency, education, and general suitability, but I have nevertheless so far failed in obtaining an inspectorship, and have no hope of ever succeeding. I only mention my own case as a type of probably hundreds of other women, who cannot hope for promotion, and who, I have no doubt, regret as much as I do, having entered a most arduous calling, the prizes of which go to other professions, and who must only continue to be mere drudges, in spite of years of good work, and of the experience which cannot be gained in the lecture-rooms of a medical school, or the wards of a general hospital. I hope you will pardon me for troubling you, but I thought I should like to call your attention to what can only be regarded as very unjust. Perhaps you will not consider my letter suitable for publication, but if it be published I am sure that a large number of experienced and practising midwives will heartily subscribe to my opinions.

### Appointments.

[No charge is made for announcements under this head, and we are always glad to receive, and publish, appointments. The information, to insure accuracy, should be sent from the nurses themselves, and we cannot undertake to correct official announcements which may happen to be inaccurate. It is essential that in all cases the school of training should be given.]

ADELAIDE HOSPITAL, SOUTH AUSTRALIA.—Miss M. Walsh has been appointed charge-nurse. She was trained at the Adelaide Hospital, and has since been charge-nurse at the Mount Gambier Hospital.

AHMEDNAGAR, INDIA.—Miss Alice A. Bowles has been appointed, by the Society for the Propagation of the Gospel, missionary nurse. She was trained at the Homœopathic Hospital, Great Ormond Street, London; the City of London Lying-in Hospital; and Queen Victoria's Jubilee Institute, Edge Hill, Liverpool, for district nursing.

BOLINGBROKE HOSPITAL, WANDSWORTH COMMON.—Miss Theresa Russell has been appointed matron. She was trained at St. Thomas's Hospital, London, where she was subsequently sister. She has since been matron at Bromsgrove Isolation Hospital.

BRENTFORD UNION INFIRMARY, ISLEWORTH.—Miss Ethel Cooling and Miss E. Bowdidge have been appointed sisters. Miss Cooling was trained at the Brentford Union Infirmary; she was subsequently on the staff of the Mildmay Deaconess Association, sister at St. Luke's Hospital, Halifax, which she left to return to her training school as pupil midwife. She holds the L.O.S. certificate. Miss Bowdidge was trained at the Southwark Infirmary, East Dulwich.

CAWNPORE STATION, INDIA.—Miss F. A. Pitman has been appointed, by the Society for the Propagation of the Gospel, missionary nurse. She was trained at Guy's Hospital, London, and holds the medal for five years' service. She has been midwifery sister at the Bristol Royal Infirmary.

CITY HOSPITAL, WALKER GATE, NEWCASTLE-ON-TYNE.—Miss M. B. Vickers has been appointed night superintendent. She was trained at the Royal Infirmary, Bradford, where she afterwards became sister, and she has since been sister at the Children's Hospital, Bradford.

CONSUMPTIVES' HOME, NORTH TERRACE, ADELAIDE, SOUTH AUSTRALIA.—Miss Norman has been appointed matron, and Miss Hamp charge-nurse. Miss Norman was trained at the Melbourne Hospital and has since been charge-nurse at the Adelaide Hospital. Miss Hamp was trained at the Adelaide Hospital.

DISTRICT AND DISPENSARY HOSPITAL, SELBY. — Miss Mooney has been appointed matron. She was trained at Limerick Hospital and at Cork Street Fever Hospital, Dublin. She has since been in charge of the Stanningley and Farsley Nursing Districts.

EXMOUTH COTTAGE HOSPITAL.—Miss Alice E. Seaton has been appointed matron. She was trained at the Brentford Union Infirmary, Isleworth. She was subsequently district nurse at Acton and sister at the Infirmary, Isleworth. She holds the certificate of the Incorporated Society of Trained Masseuses.

ISOLATION HOSPITAL, BARROW-IN-FURNESS.—Miss Lizzie Muir has been appointed charge-nurse. She was trained at the City Hospital, Edinburgh, and the County Hospital, Lincoln. She has since been charge-nurse at Edinburgh City Poorhouse Hospital, matron of the Port of London Sanitary Hospital, and has done private nursing.

MOUNT GAMBIER HOSPITAL, SOUTH AUSTRALIA.—Miss Lena McLean has been appointed charge nurse. She was trained at the Adelaide Hospital.

NORTHERN HOSPITAL, MANCHESTER.—Miss Joyce Simonds has been appointed sister. She was trained at the Clinical Hospital, Manchester, and at the London Hospital, Whitechapel.

PORT PIRIE HOSPITAL, SOUTH AUSTRALIA.—Miss Alice Pizey has been appointed charge nurse. She was trained at the Adelaide Hospital.

RAWCLIFFE HOSPITAL, CHORLEY.—Miss Margaret Brown has been appointed staff nurse. She was trained at West Norfolk and Lynn Hospital, King's Lynn, and has since been charge nurse at Wrexham Infirmary.

ROYAL CITY OF DUBLIN HOSPITAL.—Miss Milly Hamilton has been appointed staff sister. She was trained at the Samaritan Hospital, Belfast, and the Royal City of Dublin Hospital. She has since been home sister at the Nurses' Home attached to the Royal City of Dublin Hospital.

ST. MARK'S HOSPITAL FOR FISTULA, CITY ROAD, LONDON.—Miss Ida Gillett has been appointed sister. She was trained at the Croydon General Hospital, and has since been sister at the Victoria Hospital, Blackpool.

THE SANATORIUM, CHARTERHOUSE SCHOOL.—Miss A. L. Johnson has been appointed matron. She was trained at Southwark Infirmary and the North-Western Fever Hospital. She has since done private nursing in Godalming and frequently at the Charterhouse School.

WICK FEVER HOSPITAL.—Miss Christian McGillvray has been appointed superintendent nurse. She was trained at the Fever Hospital, Sunderland, and has been charge nurse at the County Fever Hospital, Ayr, Leith Small-pox Hospital, Knightswood Hospital, and Belvedere Hospital, Glasgow.

### Echoes from the Outside World.

### Movements of Royalty.

THE KING, who with the Queen, is to open Parliament in person on February 14th, left London for Sandringham on Saturday. The Chevalier de Martino, his Majesty's marine painter, was on the platform at St. Pancras, when he arrived, and conversed with him before his Majesty entered the saloon. The King and Queen leave Sandringham on Saturday for Windsor Castle, where they will reside until the removal of the Court to Buckingham Palace. On Friday, February 7th, they will hold a Diplomatic and Official Court at 10 P.M., and a Court on Friday, February 14th, at 10 P.M. The King has commanded that the Court shall wear mourning for fourteen days from January 18th for her late Royal Highness the Grand Duchess of Saxe-Weimar-Eisenach.

### The King and the Church Army.

THE King commanded the presence at Buckingham Palace on Friday of the Rev. Wilson Carlile, head of the Church Army, who, at the request of his Majesty, gave an account of the work of the various departments of the organisation. According to the official report, the King informed Mr. Carlile that he had heard so much of the good work of the Church Army from the Duke of Fife and others that he wished it every possible success. He was glad to think that so many married men with families were assisted by labour who could not otherwise obtain relief from the boroughs for lack of the usual six months' residence. He laid stress on the importance of work as the society's great test for sincerity, preventing the loafer from imposing upon the public, and discouraging men from being attracted from the country to the metropolis. In conclusion, his Majesty asked Mr. Carlile to convey the following message to the members of the Church Army:—"Give your devoted workers my deepest sympathy. Encourage them to press on and to persevere. I also send my deepest sympathy and encouragement to the poor inmates of your homes, who, I hope, will show gratitude for the benefits received." After Mr. Carlile had left, the King sent him by Colonel Legge, his equerry, a bank-note for £100, with the hope that "it might be helpful to some of England's poorest, and that the necessary means for the maintenance of the work would be forthcoming."

### The War in the Far East.

THE Tsar has issued an Order of the Day to his army and navy, in which he says that Port Arthur has fallen into the hands of the enemy after a siege of eleven months. His Majesty pays a tribute to the heroism of the garrison, and, observing that the enemy is bold and strong, avers that the struggle with him at a distance of 10,000 versts from the sources of Russian strength is "indescribably hard." He adds, however, "But Russia is powerful. In the thousand years of her life there have been still harder trials, from which she has always emerged stronger." With all Russia, the Tsar trusts that the hour of victory will soon dawn.—The transfer of Port Arthur was completed on January 10th, and it is stated that there were no signs of privation. But General Stössel, in bidding farewell to his garrison, and thanking them for their heroic defence, declared that he would take upon himself all blame for a premature surrender. —General Nogi has forwarded a message to the friends of Japan in England. He says, knowing the sympathy of the people of Great Britain, he and his soldiers heartily rejoice; and as representing the Army, he sends heartfelt thanks.— The Kaiser has notified to Generals Nogi and Stössel his intention of conferring on them the Order Pour le Mérite, and they have returned their warm thanks for the honour.

### Woman's Work.

THE Poet Laureate, Mr. Alfred Austin, gave an address on Thursday last week to the Girls' Realm Guild of Service and Good Fellowship, in the course of which he said: "These were days when we were all brought up to think, and I still believe, that women should be the trustees of domestic virtue, the exemplars of refined manners, the guardians of delicacy of thought and action, and I can see no reason to abandon that creed for one that appears to be more popular in an age supposed by itself at least, to be peculiarly progressive. I have sometimes thought that, for the noblest women, the Three Graces are those of Faith, Hope, and Charity; steadfast belief in the goodness of good, unflickering hope of moral and spiritual betterment, and inextinguishable love in the widest signification of that sacred word." Referring to personal service offered by the individual to the individual, which Mr. Austin described as the central aim of the Guild, he affirmed that he could conceive no nobler mission, and he urged his hearers never to desist from taking part in it, so that they might enjoy to the end "the luxury of doing good;" compared with that luxury the sweets of society, the attainment of power which in reality was only highly-paid servitude, and even the supposed solace of unquestioned fame, were shadows, not substantial things.

### Mrs. Meynell-Ingram's Bequests.

THE will of Mrs. Meynell-Ingram, of Hoar Cross, Staffordshire, sister of Lord Halifax, which has just been proved, is remarkable for the bequests of an interesting character. For instance, she left £500 for an additional endowment to the "Meynell-Ingram Scholarship" at St. Anne's College, Abbots Bromley, £500 for the Meynell-Ingram Scholarship to the Lichfield Theological College, and £500 for an additional scholarship to be called "the Meynell-Ingram Scholarship No. 2." She bequeathed £1,000 to Canon Knox Little, Vicar of Hoar Cross, £500 to his son Hugo Charles, and for distribution among the choirmen at Hoar Cross who have been members of the choir for five years a sum of £300. To her servants she left £50 to two maids, £250 to the head gardener at Temple Newsam, her place in Yorkshire, £250 to the coachman, £100 to the head carpenter at Hoar Cross, £50 to the housekeeper, £100 to the butler, an annuity of £60 to the head gardener at Hoar Cross, an annuity of £30 to the groom, and an annuity of £26 to the laundry-maid. To "her faithful servant, James Tomlinson," she left an annuity of £50 and the use of the house in which he resides rent free, while to every workman who had been in her employ for 20 years she bequeathed £20, and to those of 10 years' service £10 each.

### Civil Service Examinations for Women.

IT is officially intimated that in March simultaneous competitive Civil Service examinations will be held in London and the principal provincial centres for women clerkships and girl clerkships in the General Post Office. Applications for permission to attend the examinations must be made not later than February 9th, and no one will be admitted to the competition who is under five feet in height. The subjects are English, composition, arithmetic, geography, Latin, French, and German, of which only two may be taken up, and English history, algebra, and shorthand, of which only two may be taken up. Every girl candidate must produce an undertaking signed by her parent or guardian to the effect that, if successful, she will reside with her parents or guardians or with relatives or friends approved by them. Girl clerks for the first two years serve six hours a day, and are paid at the rate of £35 and £37 10s. for three years respectively. If their competence is proved they may be promoted as vacancies occur in the class of women clerks, in which the hours of work are seven and the rate of pay commences at £55 per annum, increasing by annual increments to £100. There is always the possibility of promotion to higher rank and pay.

### A Book and its Story.

### SANDRINGHAM AND WEST NORFOLK.*

Mr. W. A. Dutt has produced for the Homeland Association an interesting historical survey of that particular corner of the county of Norfolk to which he has been asked to restrict his attention. It is a district, however, not lacking in materials sufficient for more than one volume, for it is rich in historic memories. Mr. Rider Haggard prefaces the book with an Introduction, which gives weight to its contents; as a resident in North-West Norfolk, the subject is one in which he takes an active interest. Of the title, he writes in his opening paragraph: "'The King's Homeland' is a large title, for wherever the flag of Britain flies—that is, over nearly one quarter of the globe—there is the homeland of King Edward VII.; and there, surely, north and south, and east and west, in snows or sands, in cities or plains, he would find himself as much at home and as much beloved as he is at Sandringham." In the mention of historic houses Sandringham will, of course, stand first in interest to all loyal subjects of the King. But the book is full of interest throughout, and we will start with an extract, by way of introduction, to its vicinity—again from Mr. Haggard's pen, "Then there is the ancient town of Lynn, standing on its flat lands near the sea—Lynn, that was a port before the Norman set foot in England, and remains a port considerably improved to-day, although, perhaps, its trade is not what it once was. Castle Rising is rich in historic associations. Its interesting old Norman keep is believed to have been built by William, first Earl of Sussex, the son of William de Albini, 'pincerna,' or butler, to William II., by whom the town and lordship were given to him. Most interesting are the remains of the castle on its mound, set within the great earthworks raised for purposes of defence by Saxon or even earlier folk. Castle Rising, even as late as the year 1832, was a place of some importance politically, for it, until that date, returned two members to Parliament." Houghton Hall, of which Mr. Haggard writes as follows, is full of pathetic suggestions in connection with the Walpole family:—"Many other are the places in North-West Norfolk that our traveller should visit. For instance, there is Houghton, Sir Robert Walpole's vast and melancholy monument. It is a huge palace and splendid in its way, but I think . . . it is easy to understand how Houghton would affect a temperament such as his. See what he writes in that melancholy letter to George Montague: 'Here I am alone at Houghton, and alone in this spot where, except for two hours last month, I have not been for sixteen years. . . . Here I am, for probably the last time of my life . . . Every clock that strikes tells me that I am an hour nearer to yonder church—that church into which I have not the courage to enter, where lies the mother on whom I doted and who doted on me! . . . There, too, lies he who founded its greatness, to contribute to whose fall Europe was embroiled—there he sleeps in quiet and dignity." The above extract from Sir Horace Walpole's letter was written during a visit to Houghton, while he was contesting an election at Lynn in March 1761. He died six years later, and with him the male line of Sir Robert Walpole became extinct. West Norfolk claims the distinction also of being the birthplace of Lord Nelson.

The greater part of the "King's Homeland" has to do with Sandringham itself. Its history from the Norman Conquest to the present day forms a record which is particularly interesting. The earliest mention of Sandringham is found in Domesday, where it figures under the name of Sant-dersingham. Mr. Dutt quotes from Blomefield's history of Norfolk: "In the time of Edward the Confessor it was held by a freeman of Harold, who was afterwards King of England. . . . Harold, as everyone knows, was killed at the Battle of Hastings, and the manor was then given to Robert Fitz-Corbun, who was lord at the time of the survey. Sandringham remained in the possession of the Fitz-Corbuns, or Curzons, until some time subsequent to the reign of Henry II. . . . and in that of Edward III. Roger de Sandringham and Alice, his wife, were interested in its lands from whom these, or some of them, passed to the Cobbe family by marriage with the heiress of the Rivets." The property appears to have remained for many centuries in the Cobbe family, and finally in 1870, after some vicissitudes, and change of owners, became the property of the King, when Prince of Wales. Of the old hall, approached by a magnificent avenue of limes planted in a double row, which was in existence long before the present Royal residence, there is now no trace. The present mansion has twice been altered and enlarged since its erection. The style of the house may be described as modified Elizabethan, and, whether approached from the east or west, its appearance is dignified and pleasing. It does not face the lime avenue, which runs north and south, for its main entrance is on the east side. Here there is a large porch with Romanesque arches, surmounted by a pierced stone parapet, and having over the eastern arch the royal arms. Over the door is an inscription in Old English letters, which relates, "This house was built by Albert Edward and Alexandra his wife, in the year of our Lord 1870." The west front, however, is the principal one, and has a frontage of some 500 feet. Perhaps the most general idea that people have of Sandringham of to-day is that of a model estate, a home to which the King retires for relaxation from State affairs, and where he and Queen Alexandra can receive their guests in an hospitable and less formal manner than in the other Royal residences. But that there are wider interests involved in its possession, readers of the "King's Homeland," will easily realise. On the "very barren soil" of the district a model farm has been raised "which might be imitated with advantage to themselves and the country by all who are sufficiently persevering, wealthy and intelligent to do so." Then other matters connected with the well-being of those employed on the estate receive particular attention. "Upon how many properties, even where the expenditure of money need not be considered, is anything to be found resembling the Sandringham Club, established for the use of all workmen above the age of fourteen years?" This, however, is not a purely charitable institution, since its members pay a subscription of one shilling a quarter, and also pay for all refreshments at the time of ordering. Attached to the recreation-room, which is provided with papers, etc., there is a surgery, the value of which all dwellers in the county will realise. The technical school established by the Queen provide instruction in copper and brass work; the weaving of tapestry and of wool of the Sandringham Southdowns into homespun; joinery and carving is also taught, and the excellence of the articles produced is evidence of the practical work of the school. "Many a Norfolk lass and lad must have reason in after life to bless the kind thought which led to their being able to earn good livelihoods as masters of their respective trades, instead of spending their lives in domestic service, or as followers of the plough." "The King's Homeland" is beautifully illustrated, and the chapters devoted to rare plants found in the Sandringham district will be an attraction to students of botany.

* "The King's Homeland." By W. A. Dutt. (A. and C. Black. 10s. net.)

### Notes and Queries.

### REGULATIONS.

**The Editor is always willing to answer in this column, without any fee, all reasonable questions, as soon as possible.**

**But the following rules must be carefully observed:—**

**1. Every communication must be accompanied by the name and address of the writer.**

**2. The question must always bear upon nursing, directly or indirectly.**

**If an answer is required by letter a fee of half-a-crown must be enclosed with the note containing the inquiry.**

*Plague Nursing in India.*

(120) Will you kindly tell me where I can obtain particulars of plague nursing in India?—*M. H.*

Write to the Under Secretary of State, India Office, S.W.

*Registration of Midwives.*

(121) Can you please inform me if I can get registered as a midwife? I am a certificated monthly nurse and have been practising as midwife for seven years.—*L. M.*

Write to the Secretary of the Central Midwives Board, 6 Suffolk Street, Pall Mall, S W. There is a book published by the Scientific Press, "How to become a Midwife" (post free, 1s. 1d.), which may be of assistance to you.

*Civil Hospitals in India.*

(122) Can you give me any information about nursing in civil hospitals in India? Also are there any nursing books on the subject?—*Guy's Nurse.*

Among the civil hospitals are:—The Howrah General Hospital, Calcutta; the Cama Hospital, Bombay; the Secunderabad Civil Hospital; the Madras General Hospital. Write to the respective matrons for information; and for full list see "Burdett's Hospitals and Charities."

*Maternity Case.*

(123) I am a maternity nurse. My case came off seven days before the time I was engaged for. I was still at a case, but I came to the patient for the first day till all was over. Can I claim any fee for that day's work?—*A. E. F.*

The one day would be included in the month's work, that is, if you were engaged for the usual month. If you did not take on the case subsequently, you may certainly claim for the work done.

*Poor Law Infirmary Training.*

(126) Will you kindly tell me whether a nurse trained under Poor Law is acting unfairly if she leaves her training school at the end of three and a half years? I have left my training school for over two years, since when I have been charge nurse in a Poor Law infirmary and engaged in private nursing. On applying recently for the post of "sister" at a Poor Law infirmary, I was informed by the Chairman that it was "disgraceful, and just like you nurses to go and be trained at the expense of ratepayers, then to take your services elsewhere."—*One Who likes Justice.*

When you had finished your three years' training you were, of course, perfectly free to go where you liked if you did not wish to remain at your first infirmary, or if there was no vacancy to enable you to do so.

*Circumcision.*

(125) One of my greatest difficulties as a maternity nurse is to persuade the parents of baby boys, when necessary, to have them circumcised. Is there any literature on the subject? The nurse is on the right side when the doctor approves of it, but there are times when it is equally necessary, and the doctor, for some incomprehensible reason, is not in favour of it.—*Sister Dora.*

When surgically necessary the medical man will do the persuading. When not imperative it is a matter entirely for the parents to decide. You cannot force your own theories, and, of course, it would be a great breach of professional etiquette to go against the medical man's opinion in any case you were nursing under him.

*Home.*

With reference to Query 119, the Nurses' Settlement Home is only open, we are requested to remind nurses, to members of the Royal British Nurses' Association who are past work, and have a small pension from the Royal National Pension Fund or some other source.

**Handbooks for Nurses.**

| | Post Free. |
|---|---|
| "The Nursing Profession: How and Where to Train" ... | 2s. 4d. |
| "The Nurses' Pronouncing Dictionary" ... ... ... | 2s. 0d. |
| "Nursing: its Theory and Practice" (Lewis) ... ... | 3s. 6d. |
| "Surgical Bandaging and Dressings" ... ... ... | 2s. 0d. |
| "A Complete Handbook of Midwifery" (Watson) ... | 6s. 4d. |

Of all booksellers or of The Scientific Press, Limited 28 & 29 Southampton Street, Strand, London, W.C.

### For Reading to the Sick.

### THE CARVER'S LESSON.

TRUST me, no mere skill of subtle tracery,
No mere practice of a dexterous hand,
Will suffice, without a hidden spirit,
That we may, or may not, understand.

And those quaint old fragments that are left us
Have their power in this—the Carver brought
Earnest care, and reverent patience, only
Worthily to clothe some noble thought.

. . . . . . . .

But I think, when years have floated onward,
And the stone is grey, and dim, and old,
And the hand forgotten that has carved it,
And the heart that dreamt it still and cold;

There may come some weary soul, o'erladen
With perplexèd struggle in his brain,
Or, it may be, fretted with life's turmoil,
Or made sore with some perpetual pain.

And the tendrils will unroll, and teach him
How to solve the problem of his pain;
And the birds' and angels' wings shake downward
On his heart a sweet and tender rain.

While he marvels at his fancy, reading
Meaning in that quaint and ancient scroll
Little guessing that the loving Carver
Left a message for his weary soul.

*A. A. Procter.*

Some of earth's fairest flowers grow where no human eye can be pleased with their loveliness. They are Nature's offering to her Creator. In some grand cathedral you may find the most exquisite bits of carving and sculpture high up above the earth, "hidden from every eye but His to Whose glory all was built." So should the Christian life be. Its best, its most beautiful side should be seen by God only. There are deeds of hidden loveliness which are the results of inward purity; there are exquisite touches of spiritua beauty which are like the crystallisation of pure thoughts too refined, too delicate for the rude touch of earth; there are delicate courtesies which just seem to touch the common life like a breath from a purer world; we are hardly aware of them, but they are very precious, they are a soul's offering, they bear the mark of the Cross; there are thoughts, and aspirations, and prayers which go Heavenward, fragrant as the perfume of some hidden flower. These all give their touch of beauty to the life as God sees it. They impart to the soul itself something of the joy of God as he rejoices in the love of His creature. No life should be quite without this sweetness for your own sake, as well as for Christ's sake and your neighbour's. No day should pass without some effort in this direction. "Let that day be judged mis-spent in which there has been no deed of hidden loveliness." This will be useful as an unwritten law of daily life, something to strive after, something to attain unto.—*Rev. J. Brett.*

Work with Him truly in life's daily task,
And what the future hides nor fear nor ask;
Seek His will only—leave to Him the rest,
And toil or suffer, as shall please Him best.

*C. M. Noel.*

